# Essential role of mitochondrial Stat3 in p38^MAPK^ mediated apoptosis under oxidative stress

**DOI:** 10.1038/s41598-017-15342-4

**Published:** 2017-11-13

**Authors:** Xinlai Cheng, Christiane Peuckert, Stefan Wölfl

**Affiliations:** 1 Institut für Pharmazie und Molekulare Biotechnologie, Ruprecht-Karls-Universität Heidelberg, Im Neuenheimer Feld 364, 69120 Heidelberg, Germany; 2 Department of Organismal Biology, Uppsala University, Uppsala, S-75236 Sweden

## Abstract

Stat3 is an oncogene, frequently associated with malignant transformation. A body of evidence implicates that phospho-Stat3^Y705^ contributes to its nucleic translocation, while phospho-Stat3^S727^ leads to the accumulation in mitochondria. Both are of importance for tumor cell proliferation. In comparison to well-characterized signaling pathways interplaying with Stat3^Y705^, little is known about Stat3^S727^. In this work, we studied the influence of Stat3 deficiency on the viability of cells exposed to H_2_O_2_ or hypoxia using siRNA and CRISPR/Cas9 genome-editing. We found dysregulation of mitochondrial activity, which was associated with excessive ROS formation and reduced mitochondrial membrane potential, and observed a synergistic effect for oxidative stress-mediated apoptosis in Stat3-KD cells or cells carrying Stat3^Y705F^, but not Stat3^S727D^, suggesting the importance of functional mitochondrial Stat3 in this context. We also found that ROS-mediated activation of ASK1/p38^MAPK^ was involved and adding antioxidants, p38^MAPK^ inhibitor, or genetic repression of ASK1 could easily rescue the cellular damage. Our finding reveals a new role of mitochondrial Stat3 in preventing ASK1/p38^MAPK^-mediated apoptosis, wich further support the notion that selective inhibition mitochondrial Stat3 could provide a primsing target for chemotherapy.

## Introduction

Inflammation plays an important role in tumor initiation and progression^1^. Signal transducer and activator of transcription 3 (Stat3) is one of seven Stat proteins and can be activated by growth factors, cytokines, and oncogenic kinases in the inflammatory microenvironment including ultraviolet radiation, carcinogenic chemicals, stress and smoking^2–7^. Stat proteins, in particular Stat3, are highly activated in a number of cancer cell lines and human tumor samples^8^. It has been shown that constitutively active Stat3, but not a dominant-negative mutant, is present in Src-associated malignant transformation^4,9^. In general, intrinsic and extrinsic factors can stimulate tyrosine kinases, which phosphorylate Stat3 at tyrosine 705 (phospho-Stat3^Y705^) to generate binding sites for SH2 domain and in turn form homo- and heterodimers with Stat3 or other Stat members^10^. Activated Stat dimers then translocate to the cell nucleus, bind to specific DNA sequences and directly regulate expression of anti-apoptotic genes, including Bcl-xl and Mcl as well as pro-survival genes, like c-myc and cyclin D1^5,11^. Phosphorylation at serine 727 (phospho-Stat3^S727^) contributes to achieve maximal activation of Stat3^12^. Recently, several reports described the importance of phospho-Stat3^S727^, but not phospho-Stat3^Y705^, for the Stat3 mitochondrial translocation^13,14^. They showed that Stat3 in mitochondria interacted with enzymes of the electron transport chain (ETC) to regulate mitochondrial oxidative phosphorylation and facilitated Ras-induced malignant transformation^13,15–17^. There is also compelling evidence that increased levels of apoptotic cells have been frequently observed in Stat3 inactive or deficient tumor cells^13,15,18^. However, the signaling pathway involved in the lack of mitochondrial Stat3-mediated apoptosis is not well elucidated yet.

p38^MAPK^, ERK (extracellular signal-regulated kinase) and JNK (c-Jun NH2-terminal kinase) belong to the mitogen-activated protein kinase (MAPK) family. In comparison to ERK and JNK, which support cell proliferation and survival, p38^MAPK^ has been widely accepted as an inhibitor of proliferation or a regulator of cell apoptosis^14,19^. p38^MAPK^ can be phosphorylated and activated by diverse upstream activators MAPK kinase kinase (MKKKs), like ASK1^20–23^. p38^MAPK^ also acts as a free radical sensor and inhibits malignant transformation and tumorigenesis by inducing cell cycle arrest and apoptosis under oxidative stress^18,23,24^.

In this article, we studied the influence of Stat3-deficiency on cellular viability and found that Stat3-knockdown using small interfering RNA or CRISPR/Cas9 (referred to as KD cells) enhanced ROS-mediated apoptosis under oxidative stress. This synergistic effect was independent of phospho-Stat3^Y705^, but depended on p38^MAPK^ activity. Chemical inhibition of p38^MAPK^ or genetic repression of ASK1 led to rescue cellular damage. Interestingly, a similar rescue effect was observed by overexpression of Stat3^Y705F^ in KD cells, but not Stat3^S727D^. In good agreement with previous results, we found that Stat3^S727^ is of importance for its localization in mitochondria. We showed that cells lacking functional Stat3^S727^ were more sensitive to oxidative stress, which depended on ASK1/p38^MAPK^. This connection between ASK1/p38^MAPK^ signaling and mitochondrial Stat3-associated cellular apoptosis demonstrated by our data further support the notion that a specific mitochondrial Stat3 inhibitor could be of interest for clinical application.

## Results

### Stat3 knockdown leads to improved sensitivity to H_2_O_2_ in HeLa cells

**Figure 1:**
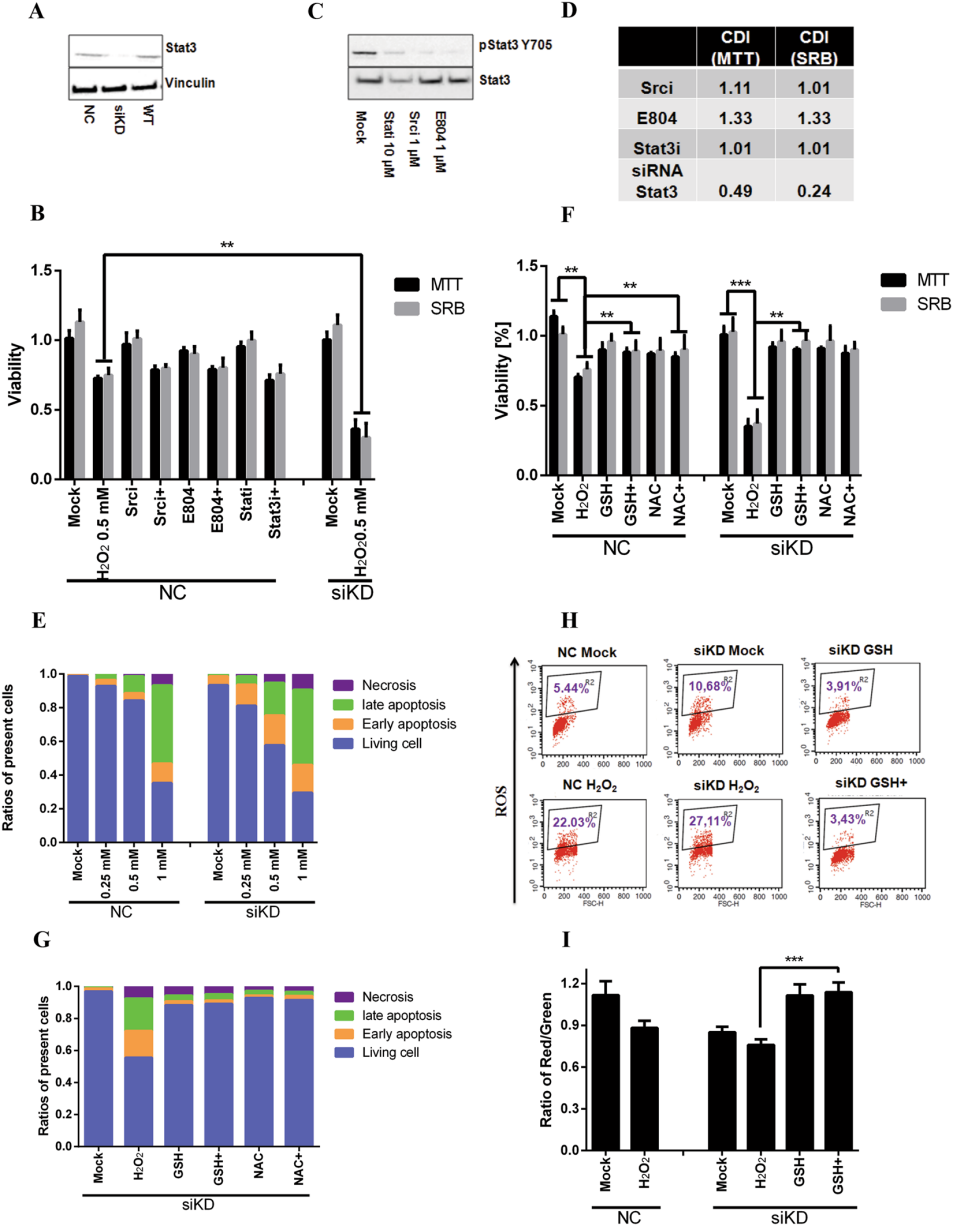
Synergistic toxic effect of H_2_O_2_ in combination with Stat3 siRNA. (**A**) The efficiency of siRNA knockdown in HeLa cells was analyzed by immunoblotting. WT: normal HeLa cells; NC: HeLa cells transfected with non-targeting siRNA. siKD: HeLa cells transfected with Stat3 siRNA. (**B**) HeLa NC and siKD cells were treated with H_2_O_2_ (0.5 mM) along, or in combination with diverse Stat3 inhibitors, including Stati (10 µM), Srci (1 µM) and E804 (1 µM) for 24 hrs. Then anti-proliferative effect was measured in MTT and SRB assays. Data were normalized to the value of non-treatment in WT cells showing the mean ± SD of quadruplicates and are representative of three independent experiments. (**C**) Inhibitory effects of Stat3 inhibitors were confirmed by immunoblotting using specific antibody against phospho-Stat3^Y705^ in HeLa cells. (**D**) Coefficient of drug interaction (CDI) was calculated for each combination as described in the experimental section. (**E**) NC or siKD cells were treated with increasing concentrations of H_2_O_2_ for 24 hrs. Annexin v/PI staining was performed to study cellular apoptosis. Apoptotic cells are annexin v-positive and necrotic cells are PI-positive. Living cells are double negative and late apoptotic cells are double positive. (**F**) Antioxidants prevented cell damage in MTT and SRB assays. NC or siKD cells were incubated with 0.5 mM H_2_O_2_ in combination with reduced glutathione (GSH, 5 mM) or N-acetyl-L-cysteine (NAC, 10 mM) for 24 hrs. (**G**) GSH and NAC rescued cell damage induced by H_2_O_2_ in apoptosis assay in siKD cells. (**H**) Elevated levels of ROS were detected in siKD cells exposed to 1 mM H_2_O_2_ for 1 hr and prevented by GSH. (**I**) Mitochondrial membrane potential was significantly reduced upon 1 mM H_2_O_2_ for 1 hr, which was compensated by adding GSH. The mitochondrial membrane potential is shown as the ratio of red-to-green floursecence stained by JC-1. + indicates in combination with H_2_O_2_. (***p < 0.001; **p < 0.01; *p < 0.05).

Stat3 is present in most human cancer cells and is frequently activated by phosphorylation at Y705, which counteracts pro-apoptotic cascades and stimulate proliferation^1^. Recent reports indicated that phospho-Stat3^Y705^ is not the only modification and phospho-Stat3^S727^ also contributes to tumor cell proliferation under oxidative stress in certain cell lines^13^. To study the role of Stat3 in oxidative stress-related cellular proliferation, we depleted Stat3 in HeLa cells by transient transfection with Stat3 siRNA (thereafter referred to as HeLa siKD cells for knockdown cells and NC cells for negative control using non-targeting siRNA). The efficiency of knockdown was more than 70% detected by immunoblotting (Fig. 1A and densitometric analysis of Stat3 expression in SI. 1). An influence of the Stat3 knockdown on cell viability was hardly detectable in 3-(4,5-Dimethylthiazol-2-yl)-2,5-diphenyltetrazolium bromide (MTT) assay and Sulforhodamine B (SRB) assay (Fig. 1B). However, upon 0.5 mM H_2_O_2_ the viability was dramatically reduced down to 40% in siKD cells, while 70% of living cells remained in NC cells (Fig. 1B).

Since phospho-Stat3^Y705^ contributes to cell proliferation^11^, we blocked the phospho-Stat3^Y705^ by recruiting three well-characterized Stat3 inhibitors, namely a Src inhibitor 4-(4′-phenoxyanilino)-6,7-dimethoxyquinazoline (Srci, Merck Src kinase inhibitor I), an in-house synthesized indirubin derivatives E804^25–28^ and the Stat3-SH2 domain inhibitor 2-hydroxy-4-(((-4-methylphenyl)sulfonyloxy)acetyl)amino)-benzoic acid (Stati, Merck Stat3 inhibitor VI). The concentrations of inhibitors were optimized to achieve a good inhibitory efficiency with a minimal toxicity (Fig. 1B,C). The inhibitory effects were confirmed by immunoblotting with a phospho-Stat3^Y705^ antibody (Fig. 1C and SI. 2). We compared the viability in HeLa NC or KD cells treated with H_2_O_2_ in the presence/absence of one of the inhibitors and calculated the coefficient of drug interaction (CDI, Fig. 1D and detail in method section). CDI showed a clear synergistic effect in the co-treatment of H_2_O_2_ with Stat3 siRNA (Fig. 1D), while an additive effect was observed in the combination with Stati, and slight antagonism in the presence of Srci and E804 (Fig. 1D).

Translocation of cytosolic phosphatidylserine (PS) from the inner to the outer leaflet of the menbrane is a hallmarker of apoptosis, which can be easily detected by fluorescent annexin v conjugate, while necrotic cells are propidium iodide (PI) positive because of the acute damage of cell membrane^23,29,30^. We treated siKD and NC cells with 0.25 mM and 0.5 mM H_2_O_2_ for 24 hr. We detected 7% and 14% annexin v positive cells in NC cells, while 17% and 37% in siKD cells (Fig. 1E and dot plots in SI. 3). Upon 1 mM H_2_O_2_ treatment only 30% cells are viable in both cell lines (Fig. 1E).

### ROS are involved in cell death induced by H_2_O_2_ in Stat3-deficient HeLa cells

ROS are crucial in H_2_O_2_-induced cell death^14,22^. In agreement with this, anti-oxidants -either N-acetyl-L-cystein (NAC) or reduced glutathione (GSH)-, easily rescued H_2_O_2_-induced cell damage in both NC and siKD HeLa cells (Fig. 1F), the number of apoptotic cells (Fig. 1G and SI. 4).

Mitochondrial Stat3 contributes to the maintenance of the mitochondrial redox homeostasis during malignant transformation and proliferation^15^. Stat3-deficiency raised ROS formation from 5% in NC to 10% in siKD cells detected with dihydroethidium (DHE)^23,29^. Adding H_2_O_2_ increased ROS level to 22% in NC and 27% in siKD cells, which was neutralized by GSH (Fig. 1H).

Since aberrant ROS could be caused by the dysregulation of mitochondrial membrane potential^23,29,31,32^, we measured changes of membrane potential using 5,5′,6,6′-tetrachlorido-1,1′,3,3′- tetraethylbenzimidazolyl-carbocyanine iodide (JC-1), a green fluorescent dye that can selectively enter into mitochondrial and at higher membrane potentials spontaneously forms red fluorescent J-aggregates^23^. Previous reports also showed that formation of the JC-1 dimer is suppressed under oxidative stress^23,29^. We found 21% reduction of the red-to-green ratio in NC cells upon hydrogen peroxide, which was further reduced in siKD cells (Fig. 1I). Adding GSH expectedly prevented membrane potential change (Fig. 1I). In summary, our results clearly demonstrated a pivotal role of ROS for enhancing apoptotic effect mediated by H_2_O_2_ in Stat3-deficienct HeLa cells.

### ASK1 activated p38^MAPK^ contributes to the synergistic effect of H_2_O_2_ induced apoptosis

**Figure 2:**
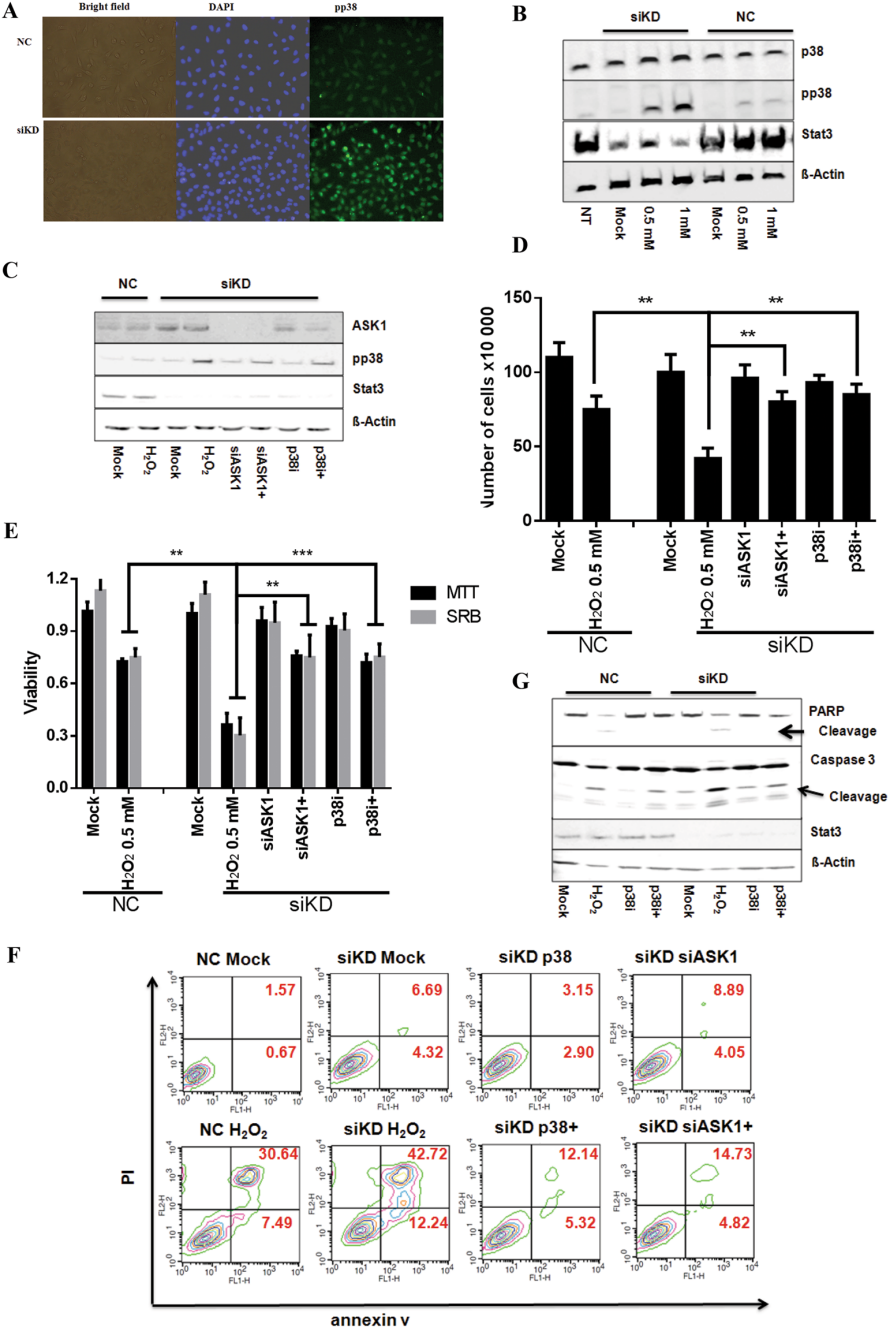
ASK1/p38^MAPK^ signaling pathway plays an important role for inducing synergistic effect in Stat3-deficient HeLa cells under oxidative stress. (**A**) p38^MAPK^ was activated in siKD cells in the presence of 1 mM H_2_O_2_ for 1 hr. Phospho-p38^MAPK^ was stained with pp38^MAPK^ antibody as green and nuclei were visualized with DAPI as blue. (**B**) Activated p38^MAPK^ was detected in siKD cells treated with H_2_O_2_. Cells (wt, NC or siKD) were treated with two concentrations of H_2_O_2_ for 1 hr. Whole cell lysates were subjected to immunoblotting and analyzed for phospho-p38^MAPK^, total p38^MAPK^ and Stat3 using respective antibodies. ß-actin was used as loading control. 40 µg proteins were loaded per lane. (**C)** ASK1 siRNA (siASK1) and p38^MAPK^ inhibitor (p38i) reduced phosphorylation of p38^MAPK^. Cells were transfected with Stat3 and ASK1 siRNA for 48 hrs. Non-targeting siRNA was used as control in NC cells. Cells were incubated with H_2_O_2_ in absence or presence of p38i for 1 hr. Whole cell lysates were analyzed by immunoblotting for expression of phospho-p38^MAPK^, ASK1, Stat3 and ß-Actin. (**D**) and (**E**) p38i or siASK1 increased cell survival. Cells were treated with 0.5 mM H_2_O_2_ in combination with p38i or siASK1 for 24 hrs. The cell growth was measured using Trypan blue (D), MTT (E) and SRB (E) assays. The data were normalized to non-targeting siRNA control, showing the mean ± SD of quadruplicates and are representative of three independent experiments. (**F**) p38i or siASK1 prevented cell apoptosis. Cells were treated with p38i or siASK1 and analyzed using annexin V/PI staining. (**G**) NC or siKD cells were treated with compounds as indicatea in the text. Whole cell lysates were analyzed for PARP, Caspase 3 and Stat3 levels. + indicates in combination with H_2_O_2_. (***p < 0.001; **p < 0.01; *p < 0.05).

Recently, several reports indicated that excessive ROS can activate p38^MAPK^-associated apoptotic cascades^18,20–23^. In comparison to NC cells, we detected increased levels of phospho-p38^MAPK^ in siKD cells exposed to H_2_O_2_ for 1 hr visualized either by immunocytochemistry (Fig. 2A) or immunoblotting (Fig. 2B and SI. 5), while total p38^MAPK^ remained constant (Fig. 2B). Recently we showed that the inactivation of ASK1 can abolish p38^MAPK^-mediated cellular apoptosis induced by Trx-R inhibitor^23^. To investigate if the ASK1/p38^MAPK^ cascade plays a role in this context, we employed SB203580, a well-known p38^MAPK^ inhibitor (p38i), or ASK1 siRNA (siASK1) published before^23^. The combination led to the reduction of H_2_O_2_-induced activation of p38^MAPK^ in siKD cells (Fig. 2C and SI. 6). We evaluated the impact of p38i and siASK1 on H_2_O_2_ mediated cell damage using trypan blue (Fig. 2D), MTT (Fig. 2E), SRB (Fig. 2E) and apoptosis assay (Fig. 2F). The results consistently showed that the inhibition of p38^MAPK^ or ASK1 knockdown could effectively prevent cell death in siKD cells. Moreover, PARP and caspase 3 are hallmarks of apoptosis^33^. We examined the cleavage of both in NC and siKD HeLa cells treated with H_2_O_2_ for 24 hrs in presence or absence of p38i. The results clearly show the enhanced cleavages of PARP and Caspase 3 in siKD cells (Fig. 2G and SI. 7), which could be prevented by p38i (Fig. 2G).

### Phospho-S727 Stat3 localizes in mitochondria

**Figure 3:**
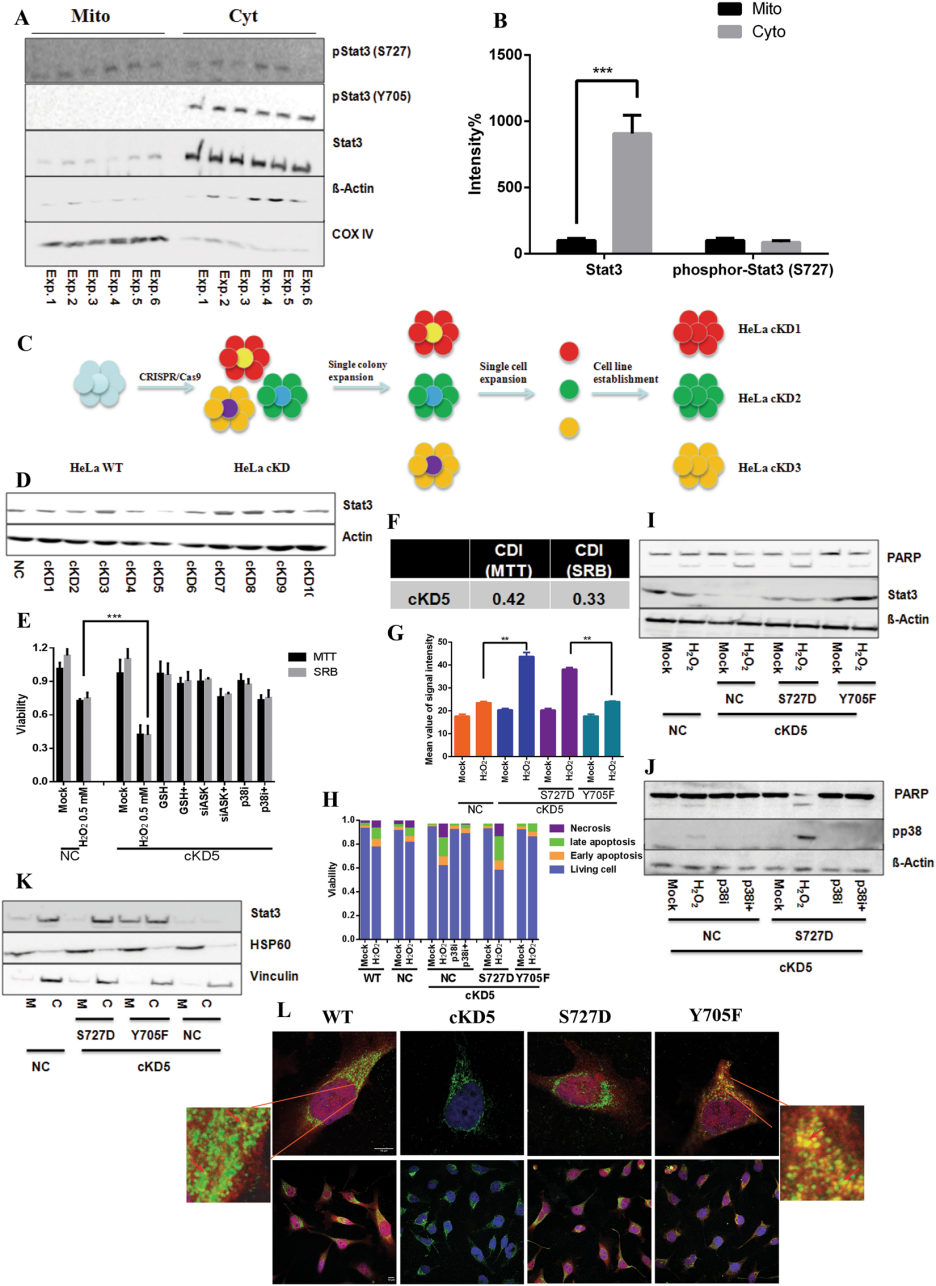
Loss of functional mitochondrial Stat3 is essential to enhance cell sensibility to oxidative stress. (**A**) and (**B**) Mitochondrial and cytosolic fractions were isolated from HeLa cells to compare the concentrations of Stat3, phospho-Stat3^Y705^ and phospho-Stat3^S727^ by immunoblotting in six independent experiments. Signal intensities were quantified and normalized to corresopnding proteins in the mitochondrial fraction. ß-actin and COX IV were used for loading control. (**C**) Schematic illustration: the generation of CRISPR/Cas9 mediated Stat3 knockdown cell lines (HeLa cKDs) with signle cell expansion. (**D**) The knockdown effect of HeLa cKDs. (**E**) GSH, ASK siRNA (siASK) and p38 inhibitor (p38i) sufficiently blocked the synergistic toxic effect by H_2_O_2_ in cKD5 cells measure by MTT and SRB assays. (**F**) Coefficient of drug interaction (CDI) was calculated. (**G**) Overexpression of Stat3^Y705F^, but not Stat3^S727D^ rescued H_2_O_2_-induced ROS formation in cKD5 cells. (**H**) Adding p38 inhibitor (p38i) and overexpression of Stat3^Y705F^, but not Stat3^S727D^ prevented cellular apoptosis induced by H_2_O_2_ in cKD5 cells. (**I**) Overexpression of Stat3^Y705F^, but not Stat3^S727D^ blocked PARP cleavage induced by H_2_O_2_ in cKD5 cells. (**J**) Adding p38 inhibitor (p38i) sufficiently inhibited phospho-p38^MAPK^ and cleavage of PARP induced by H_2_O_2_ in cKD5 cells with/without overexpression of Stat3^S727D^. (**K**) Localization of Stat3 in HeLa cells expression various genetic modified Stat3. M: mitochondrial Stat3, C: cytosolic Stat3. HSP60 and Vincublin was used as marker for mitochondrion and cytosol respectively. (**L**) Co-localization of Stat3 (red) and mitochondrial marker HSP60 (green) in HeLa WT and Stat3^Y705F^, but not Stat3^S727D^, using confocal microscope. The expression level of Stat3 in cKD cells is undetectable. Hoechst dye was used to indicate nuclear. Arrow: co-locolization of Stat3 and HSP60. Scale bar: 10 µm. + indicates in combination with H_2_O_2_. (***p < 0.001; **p < 0.01; *p < 0.05).

Recently, Larner and Levy groups described that phospho-Stat3^S727^, but not phospho-Stat3^Y705^, led to the accumulation of Stat3 in the mitochondrion^13,15^. They also showed that mitochondrial Stat3 contributed to the activity of ETC as well as to tumor initiation and progression^13,15^. Our above results showed that the suppression of phospho-Stat3^Y705^ by chemical inhibitors was not able to mimic the effect arising in KD cells. We hypothesized that mitochondrial Stat3 might play a crucial role in our observed ASK1/p38^MAPK^-mediated KD cell apoptosis. Hence we prepared cytosolic and mitochondrial extracts from HeLa cells and compared levels of total Stat3, phospho-Stat3^Y705^ and phospho-Stat3^S727^ (Fig. 3A). In agreement to previous results^15^, only a trace of mitochondrial Stat3 (10% in comparison to cytosolic Stat3) was detected in HeLa cells, while phospho-Stat3^Y705^ was undetectable in mitochondria. The level of phospho-Stat3^S727^ in mitochondria was comparable to that in cytoplasm (Fig. 3B), suggesting a crucial role of phosphorylation at S727 for localization of Stat3 in mitochondria^15^.

### Stat3^S727^ plays an essential role in p38^MAPK^-mediated apoptosis

We generated HeLa Stat3 KD cell lines using a commercially available CRISPR/Cas9 plasmid. A number of studies indicate that off-target effect of Cas9 depends on both sgRNA sequence and experimental conditions^34,35^. To obtain genetically homologous knockdown cell lines, we established 10 HeLa Stat3 CRISPR/Cas9 KD cell lines (cKDs) using single cell expansion as illustrated in Fig. 3C (detailed description in the method section). The knockdown effect was examined by immunoblotting (Fig. 3D). Among them, cKD5 showed the best knockdown effect (70%, SI. 8 and 9) and therefore was selected for our further experiments.

In good agreement to published findings^13,15^, we found less mitochondrial activity in cKD cells than in NC cells by measuring the oxygen consumption (SI. 10)^36^. We performed MTT assay (Fig. 3E), SRB assay (Fig. 3E), calculated CDI (Fig. 3F), measured ROS formation using DHE and Mitosox (Fig. 3G and SI. 11), compared mitochondrial membrane potential detected by TMRE (SI. 12) and quantified apoptotic cells (Fig. 3H and SI. 13) in cKD5 cells, and also monitored the mitochondrial ROS by adding Mitosox in real-time (Video 1–7). The results obtained were similar to those in KD cells, confirming the lost ability to regulate the cellular redox balance of Stat3-deficient cells, which led to activation of the ASK1/p38 cascade and consequently to apoptosis under oxidative stress (Fig. 3E,H–J and SI. 14 and 15).

To test the function of mitochondrial Stat3 in this context, we overexpressed Stat3 with either S727D mutation (Stat3^S727D^) or Y705F mutation (Stat3^Y705F^) in cKD5 cells. The results show that excessive ROS formation under oxidative stress could be compensated by ectopic expression of Stat3^Y705F^, but not Stat3^S727D^ (Fig. 3G). As a result, the reduction of annexin v positive cells (Fig. 3H and SI. 13) and PARP cleavage (Fig. 3I and SI. 14) was observed, showing a p38-dependend manner (Fig. 3I,J). To confirm mitochondrial localization, we purified mitochondria from cells and directly analyzed the levels of mitochondrial Stat3, which confirmed that S727 was essential for mitochondrial location of Stat3 (Fig. 3K and SI. 16). Moreover, immunostaining with anti-Stat3 antibody (Fig. 3L, red) and mitochondria specific anti-HSP60 antibody (Fig. 3L, green) showed a co-locolization of Stat3 and HSP60 in mitochondria in HeLa wt, but not Stat3^S727D^, while Stat3 was undetectable in cKD5 cells (Fig. 3L). The signal became much clearly in the case of Stat3^Y705F^.

### Stat3-deficiency related p38^MAPK^ activation in HEK293

**Figure 4:**
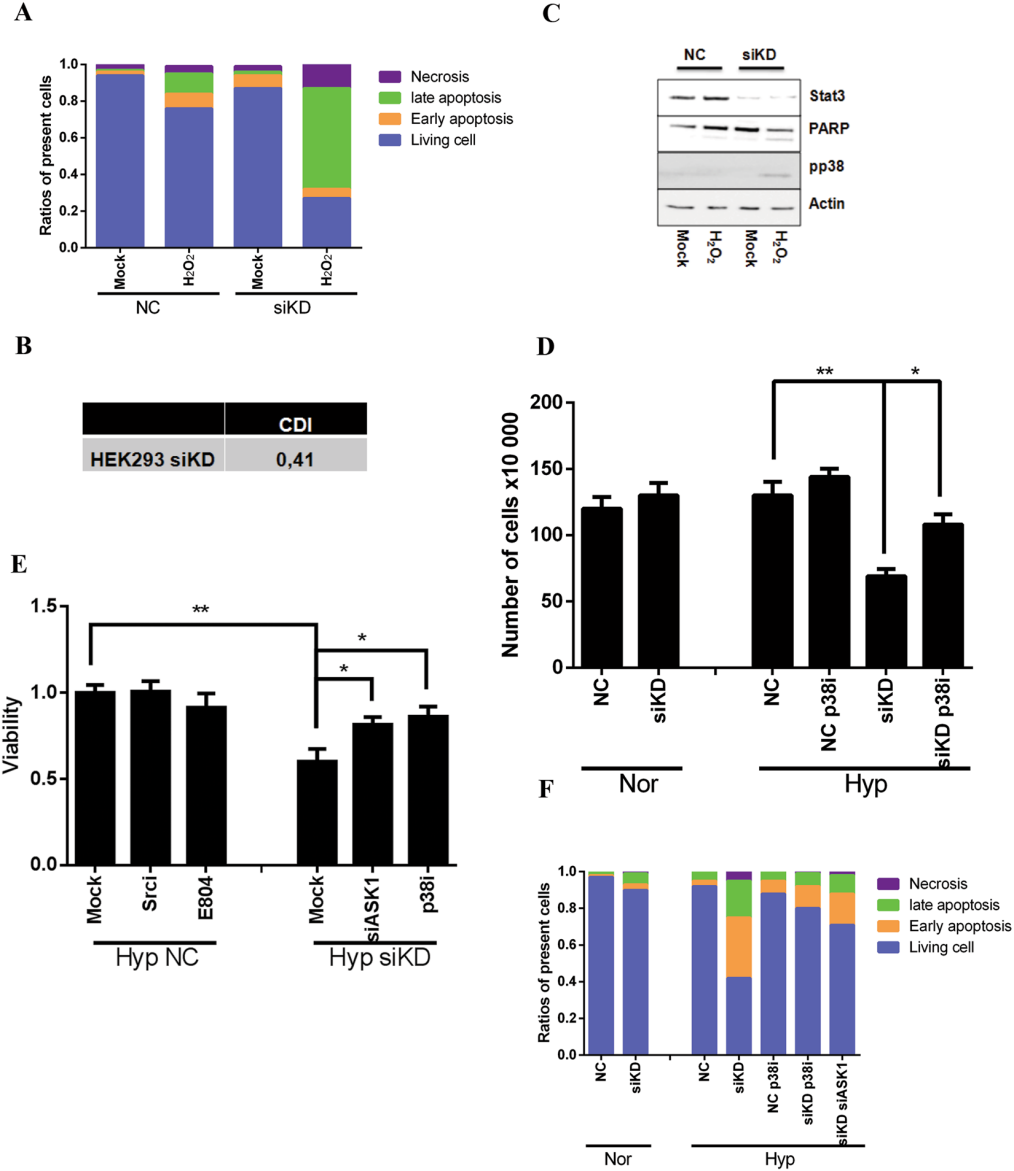
The synergestic effect is reproducible in Stat3-deficient HEK293 cells and in HeLa KD cells under hypoxia. (**A**) Enhanced apoptotic effect was observed in HEK293 transfected with Stat3 siRNA. Cells were treated with H_2_O_2_ for 24 hrs and analyzed by apoptosis assay. Apoptosis assays were performed following treatment. (**B**) Coefficient of drug interaction. (**C)** Stat3 deficiency contributed to hyperactive phosphorylation of p38^MAPK^ in the presence of H_2_O_2_. Cells were treated with 1 mM H2O2 for 1 hr. The whole cell lysate was subjected to immunoblotting. (**D**) Cells were counted after 24 hrs treatment with or without p38i in HeLa siKD cells under normoxic or hypoxic using trypan blue assay. Nor: normoxia; Hyp: hypoxia. (**E**) MTT assay was performed to evaluate the anti-proliferative effect of small molecules (p38i) or ASK siRNA (siASK) as indicated under hypoxia in HeLa siKD cells. (**F**) The influence of p38i and siASK on cell apoptosis under hypoxia was measured by annexin v/PI assay. (**p < 0.01; *p < 0.05).

Next we tested if the synergistic effect appears in HEK293 cells using Stat3 siRNA (HEK293 siKD). As shown in Fig. 4A and SI. 17, 20% annexin v positive cells could be detected in NC cells upon 0.5 mM H_2_O_2_, while 70% were found in HEK293 siKD cells. The calculated CDI was comparable to that in HeLa KD cells (Fig. 4B). Analyzing the level of Stat3 expression and activity of p38^MAPK^ by immunoblotting, we confirmed the suppressed Stat3 expression and activation of p38^MAPK^ in HEK293 siKD cells after exposure to H_2_O_2_ (Fig. 4C).

### siKD HeLa cells are more sensitive to hypoxia

To investigate whether this synergistic effect is reproducible under hypoxia, we cultivated HeLa cells under 1% oxygen^13^ and analyzed the cellular damage by trypan blue staining (Fig. 4D), MTT (Fig. 4E) and apoptosis assays (Fig. 4F and SI. 18) as described above. The viability of NC cells under normoxia and hypoxia was similar, which was reduced to 60% in siKD cells under hypoxia (Fig. 4D,E). Combination with p38i or siASK1 significantly increased cell survival up to 80% (Fig. 4D,E), in good agreement with our former results that inhibition of ASK1/p38^MAPK^ cascade prevented the synergistic effect under oxidative stress in the absence of Stat3. FACS analysis also confirmed that hypoxia induced more than 50% apoptotic cells in siKD cells, which was clearly inhibited by p38i or siASK1 (Fig. 4F).

## Discussion

The evidence that most of liver and gastric tumors originate from chronic inflammation commonly induced by infections with hepatitis B virus and hepatitis C virus, supports the concern that inflammation facilitates and promotes tumor initiation and progression^3^. In spite of the involvement of numerous genes and proteins, Stat3, a transcription factor, has been identified as an essential element in inflammation-associated carcinogenesis. Thus, the strategy to target Stat3 signaling may be beneficial for cancer therapy^5,9,37^. However, the failure to develop Stat3 inhibitors for clinical use implies that a deeper understanding of the molecular basis of Stat3 signaling is required^38^.

In the present study, we found that knockdown of Stat3 using siRNA or CRISPR/Cas9 enhanced apoptotic effect under oxidative stress induced by either H_2_O_2_ or hypoxia. In general, active Stat3 is associated with tumor cell proliferation^1,8^. In the canonical Stat3 cascade, phospho-Stat3^Y705^ is important for Stat3 translocation into nuclei to regulate downstream gene expression^1,23,39,40^. We selected three structural distinct inhibitors of canonical Stat3 signaling, but none of them could synergistically inhibit cell growth in combination with H_2_O_2_. Apparently, transcription activity of Stat3 is not involved in the synergistic effect of apoptosis induced by oxidative stress in cells lacking Stat3.

Recently, Wegrzyn *et al*. reported the identification of a trace of phospho-S727 Stat3 in mitochondria, which plays an important role in the maintenance of mitochondrial activity and suppression of ROS release from the ETC^15,41^. Moreover, mitochondrial Stat3-mediated accumulation of ROS promoted breast cancer growth^42^. In good agreement, our results showed accumulation of ROS levels and reduction of mitochondrial membrane potential in KD cells. We detected abundant phospho-Stat3^S727^ in the mitochondrial fraction. Overexpression of Stat^Y705F^ in KD cells neutralized excessive ROS and rescued cellular apoptosis in KD cells, but not Stat3^S727D^, which implicated an important role of functional S727 in regulation of cellular redox homeostasis.

MAPKs signaling is one of the most well-understood signaling processes involved in tumorigenesis^43–45^. In comparison to other MAPKs, whose functions in cell growth are still controversial, p38^MAPK^ has been identified mostly as a pro-apoptotic protein^19,46–48^, which can be activated by inflammation, environmental and genotoxic stresses^49,50^. In Ras driven cell transformation, p38^MAPK^ can be activated by increased ROS and in turn attenuate malignant transformation, while inhibition of p38^MAPK^ leads to ROS abundance and initiation/progress of tumor^19,51^. In KD cells, a clear hyperactive p38^MAPK^ was detected. Inhibiting p38^MAPK^ with chemicals or anti-oxidants interfered with peroxide-induced cell death, suggesting that the synergistic toxic effect is associated with ROS-mediated active p38^MAPK^.

ASK1 has been described as an activator of p38^MAPK^ involved in cell apoptosis induced by ROS^22,52^. Under oxidative stress, binding of Trx to ASK1 is disrupted by oxidization of Trx leading to the release and thereby activation of ASK1. ASK1 can then phosphorylate MKK3 and its downstream kinase p38^MAPK^ to induce cell apoptosis^19,23,51,53^. As expected, knockdown of ASK1 by using siRNA not only reduced the level of phospho-p38^MAPK^, but also protected cells from damage under oxidative stress and mimicked the effect of p38i in KD cells, supporting our hypothesis that Stat3-deficiency acts synergistic with the ASK1/p38^MAPK^ cascade in the induction of apoptosis.

Taken together, our results demonstrate that knockdown of Stat3 conferred cells more sensitivity under oxidative stress, most likely due to the lack of mitochondrial Stat3, which is of importance to maintain mitochondrial activity. Knockdown of Stat3 significantly reduced the malignant transformation and tumor proliferation, and induced cell damage *in vitro* and *in vivo*^13,15,41^. However, little is known about the associated molecular targets. Our findings clearly indicate that apoptosis associated with the lack of mitochondrial Stat3 was mediated by the ASK1/p38^MAPK^ cascade. Our results imply that the development of inhibitors targeting the mitochondrial activity of Stat3 could be of clinical interest as potential anti-tumor agents and could work either by interference with phosphorylation at S727 or by targeting Stat3 for degradation.

## Materials and Methods

### Materials

E804 was synthesized as previously described^25^. Structure and purity were ascertained by^13^C- and^1^H-NMR spectroscopy and elemental analyses. N-acetyl-L-cysteine (NAC), glutathione (GSH) and p38 inhibitor (p38i, SB203580) were purchased by Sigma-Aldrich (Germany). p38 (cat: #9212), pp38 (T180/Y182, cat: #9215), Stat3, pStat3 (Y705), pStat3 (S727), Caspase 3 and COX IV were obtained from cell signaling (NEB, Germany). ASK1 (cat: sc-7931) and Actin were from Santa Cruz (Germany).

### Cell culture

HeLa and HEK293 cells were cultivated in DMEM with 10% FBS and 1% Pen/Strp (PS) under 5% CO_2_ at 37 °C in a humidified atmosphere and treated with compounds as indicated. siRNA oligonucleotides were synthesized by riboxx as previously reported^54^. Sequence of ASK1 siRNA (siASK1) was reported previously^23^. Riboxx®FECT reagent was used to increase the transfection efficiency. After 48 h cells were incubated with compounds as indicated. Hypoxia (1% O_2_) was generated by Thermo Scientific Heraeus® Cytoperm® CO2/O2 Incubator as reported^29^, The cells were plated in normoxia condition for 24 hrs prior to transfection and were immediately placed in hypoxia incubator after transfection. All of the treatments were conducted within 1 hr after 48 hrs transfection and re-placed in hypoxia condition directly after treatment. All the assays were performed 24 hrs later in normoxia condition. CRISPR/Cas9 knockdown cell line was generated by using commercially available CRISPR/Cas9 plasmid (Santa Cruz, Germany). The transfection was performed as previously described using Lipofectamine 3000 (Life Technologies, Germany). 1 µg/mL puromycin was used to select transfected cells. Three days later, 10 colonies were selected manually under microscope and each was re-plated in a well of 6-well plate for 5 days. 10–100 cells of each sub-colony were isolated by using a plastic scraper. After re-plating with single cell suspension, medium was changed after 3 hrs. The places seeded with single cells or after removal of other cells were marked under microscope. The cell lines from each colony developed from single cells were isolated and established in medium without puromycin. Stat3^S727D^ (Addgene 73364) and Stat3^Y705F^ (Addgene 74434) plasmids were reported previously^55^ and purchased from Addgene. Isolation of mitochondrial and cytosolic Stat3 was described as previously reported^56^.

### Trypan blue assay

The cells were cultivated in DMEM (10% FBS), transfected with siRNAs and treated with compounds. The cells were trypsinized and re-suspended in medium. A mixture of 20 µL cell suspension and 20 µL trypan blue was added into a hemocytometer chamber^29^. The number of cells was scored under microscopy. The absolute values were listed.

### 3-(4,5-Dimethylthiazol-2-yl)-2,5-diphenyltetrazolium bromide assay (MTT assay) and Sulforhodamine B (SRB) assay

MTT and SRB assays were performed to determine anti-proliferative effect of compounds as reported^23^. Briefly, after 24 hrs seeded in 96-well plates, the transfection was performed for 48 hrs in DMEM containing 10% FCS and thereafter the cells were treated for 24 hrs in quadruplicate. The medium was removed and a solution of MTT was added for MTT assay, incubated for 1 h and quantified photometrically at 560 nm in DMSO. For SRB assay, trichloroacetic acid (50% solution) was added to stop the incubation (1 h at 4 °C). Plates were washed with water and dried overnight. A solution of 0.4% sulforhodamine B was added to stain proteins and washed with 1% acetic acid. The dye was solved with Tris buffer (10 mM, pH = 10.5) and quantified photometrically at 560 nm.

### ROS Formation assay

Cells were trypsinized, centrifuged at 200 g (1500 rpm), resuspended in FACS buffer containing 1% BSA in D-PBS and incubated with 5 µM dihydroethidium (DHE, Sigma-Aldrich), or Mitosox at room temperature in the dark for 15 min and then immediately analyzed by Fluorescence-activated cell sorting (FACS) using a FACSCalibur (Becton Dickinson) and CellQuest Pro (BD) analysis software. Excitation and emission settings were 488 nm and 564–606 nm (FL2 filter), respectively^29^.

### Apoptosis assay

Cells were cultured in DMEM, transfected with siRNAs for 48 hrs and incubated with compounds. The harvested cells were resuspended in 50 µL annexin V binding buffer, incubated with 5 µL FITC-conjugated annexin V (BD Bioscience, Germany) for 15 min in the dark at room temperature. Afterwards, 450 µL annexin V binding buffer containing 1.25 µL propidium iodide (PI, 1 mg/mL) were added, and incubation continued for 10 min in the dark at room temperature before analyzis by FACS^29^.

### Membrane potential measurement and oxygen consumption

Cells were incubated with 500 nM JC-1 (5,5,6,6-tetrachloro-1,1,3,3- tetraethylbenzimidazolylcarbocyanineiodide, Sigma-Aldrich) or 100 nM TMRE (Santa Cruz) for 15 min at 37 °C. Afterwards, cells were harvested and analyzed by FACS. Excitation and emission settings were 488 nm, 515–545 nm (FL1 channel) for JC monomers, and 564–606 nm (FL2 channel) for JC aggregates^23^. The oxygen consumption measurement was performed as our previously reported^36^.

### Immunoblotting and immunostaining

Cells were collected in urea-lysis buffer (1 mM EDTA, 0.5% Triton X-100, 5 mM NaF, 6 M Urea, 1 mM Na_3_VO_4_, 10 µg/mL Pepstatin, 100 µM PMSF and 3 µg/mL Aprotinin in PBS). Enhanced chemiluminescence (ECL) immunoblotting analysis was used and 40 µg of total protein were resolved on 8–10% SDS-PAGE gels and immunoblotted with specific antibodies as described previoiusly^57,58^.

### Imaging

Confocal fluorescent images were acquired using a TSC SP5 confocal microscope (Leica), a HCX PL APO 63x/1.3 GLYC CORR objective and LAS5 microscope imaging software (Leica). For image processing, Fiji/ImageJ was used.

### Evaluation of combinatory effects

Based on the method for calculation the coefficient of drug interaction (CDI) the combined effects of Stat3-depletion and oxidative stress were calculated. The effect of combination was calculated as follows: CDI = AB/(A × B), in which AB indicates viability of the combination; A and B indicate cell viabilities of the respective single treatment. CDI < 1 indicates a synergistic effect, CDI < 0.7 indicates a significant synergistic effect; CDI = 1 an additive effect; CDI > 1 an antagonistic effect.

### Statistical analysis

The statistical significance of compared measurements was performed using the Student’s one-tailed or two-tailed t-test (Microsoft Excel).

All data generated or analysed during this study are included in this published article.

## Electronic supplementary material

Video 1

Video 2

Video 3

Video 4

Video 5

Video 6

Video 7

Supplementary information
