# Author Correction: Essential role of mitochondrial Stat3 in p38^MAPK^ mediated apoptosis under oxidative stress

**DOI:** 10.1038/s41598-018-23431-1

**Published:** 2018-04-12

**Authors:** Xinlai Cheng, Christiane Peuckert, Stefan Wölfl

**Affiliations:** 1 Institut für Pharmazie und Molekulare Biotechnologie, Ruprecht-Karls-Universität Heidelberg, Im Neuenheimer Feld 364, 69120 Heidelberg, Germany; 2 Department of Organismal Biology, Uppsala University, Uppsala, S-75236 Sweden

Correction to: *Scientific Reports* 10.1038/s41598-017-15342-4, published online 13 November 2017

The Acknowledgements section in this Article is incomplete.

“We thank Yasamin Dabiri for her support to prepare Stat3 plasmids. This work was supported by Deutsche Forschungsgemeinschaft (CH 1690/2-1) and the BMBF grant programs Drug-iPS (FKZ 0315398B) and SysToxChip (FKZ 031A303E).”

should read:

“We thank Yasamin Dabiri for her support to prepare Stat3 plasmids. This work was supported by Deutsche Forschungsgemeinschaft (CH 1690/2-1) and the BMBF grant programs Drug-iPS (FKZ 0315398B) and SysToxChip (FKZ 031A303E).

We acknowledge the financial support of the Deutsche Forschungsgemeinschaft and Ruprecht-Karls-Universität Heidelberg within the funding programme Open Access Publishing.”

