# Supplementary material for: Essential role of mitochondrial Stat3 in p38MAPK mediated apoptosis under oxidative stress
Source: Sci Rep. 2017 Nov 13;7:15388. doi: 10.1038/s41598-017-15342-4 (PMC5684365; doi:10.1038/s41598-017-15342-4)
Supplement: Supplementary file 8 — Supplementary information [file 41598_2017_15342_MOESM8_ESM.doc]

**Essential role of mitochondrial Stat3 in p38^MAPK^ mediated apoptosis under oxidative stress**

Xinlai Cheng,^1^* Christiane Peuckert^2^ and Stefan Wölfl^1^

*Institut für Pharmazie und Molekulare Biotechnologie, Ruprecht-Karls-Universität Heidelberg, Im Neuenheimer Feld 364, 69120, Heidelberg, Germany*

*Department of Organismal Biology, Uppsala University, Uppsala S-75236, Sweden*

* Corresponding author: Xinlai Cheng: x.cheng@uni-heidelberg.de, +0049 62 21 - 54 6431

SI. 1: Densitometric analysis of Stat3 expression showed in Fig. 1A.

SI. 2: Densitometric analysis of pStat3 expression showed in Fig. 1C.


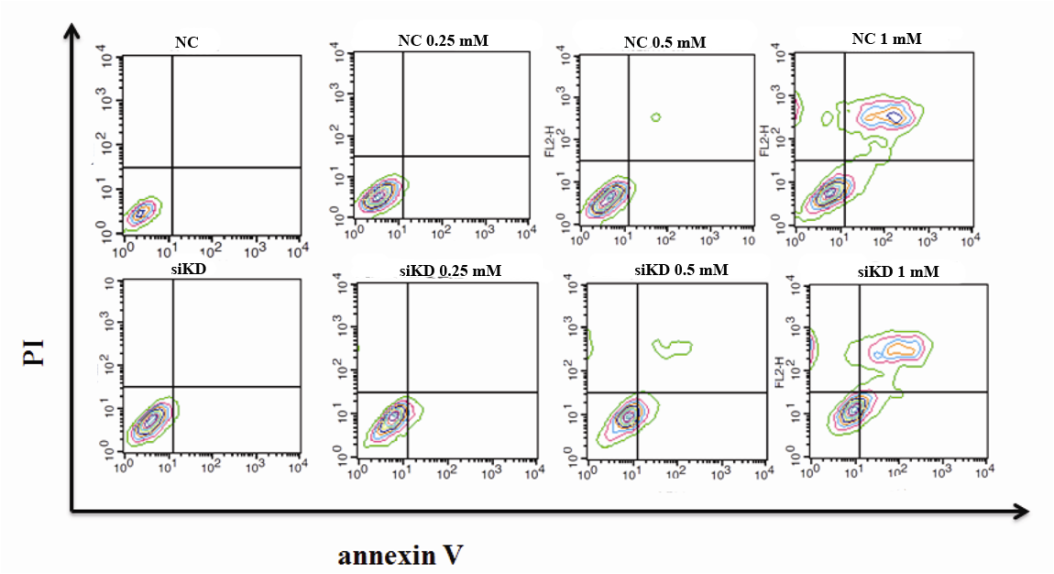


SI. 3: Dot plots of annexin v assay described in Fig. 1E


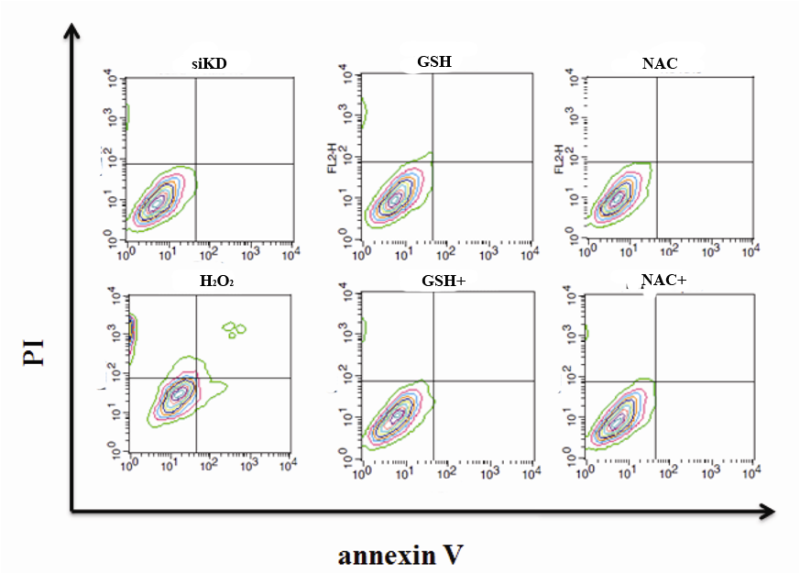


SI. 4: Dot plots of annexin v assay described in Fig. 1G

SI. 5: Densitometric analysis of pp38 expression showed in Fig. 2B.

SI. 6: Densitometric analysis of pp38 expression showed in Fig. 2C.

SI. 7: Densitometric analysis of cleaved PARP showed in Fig. 2G.


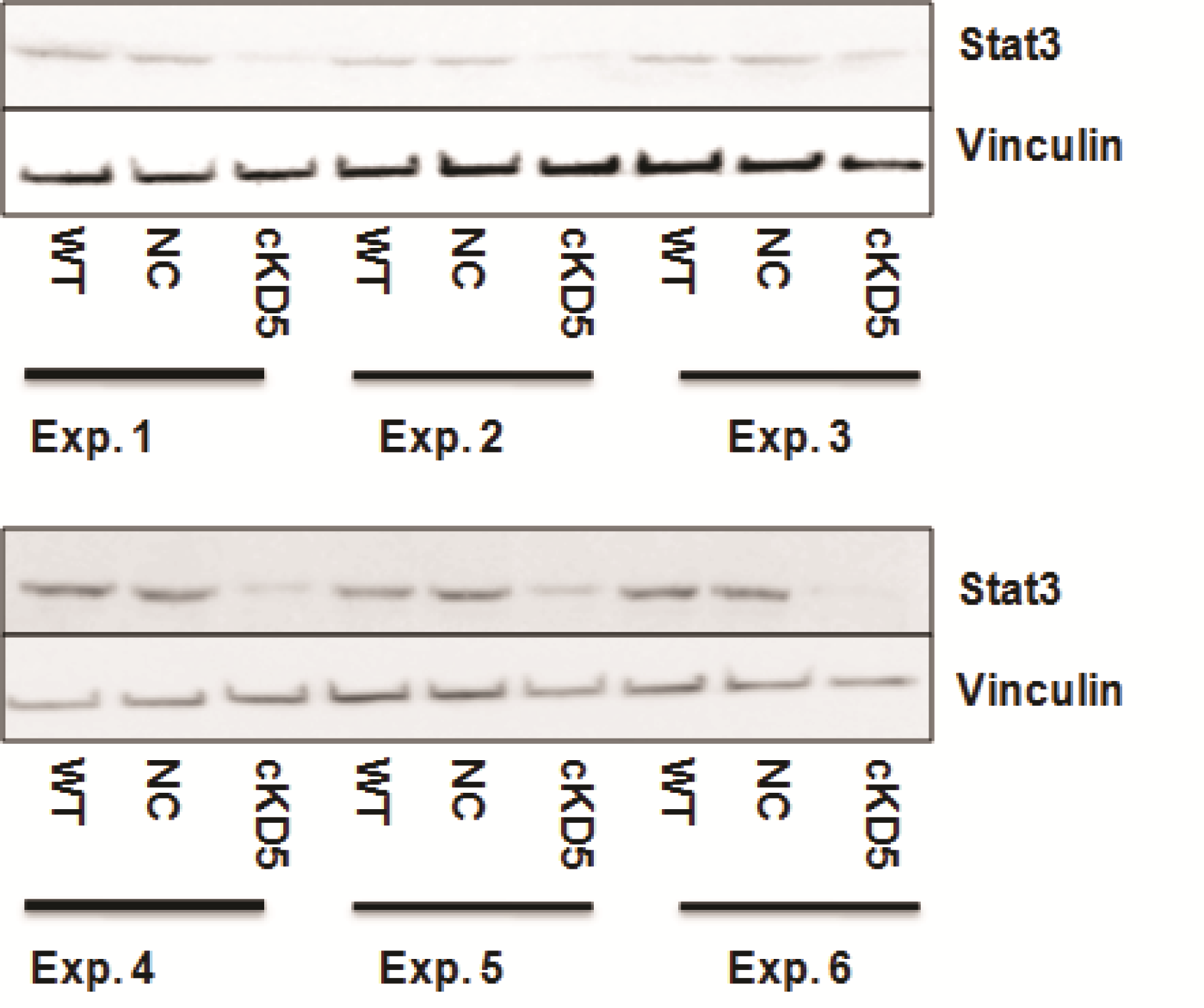


SI.8: Knockdown effect of cKD5

SI. 9: Densitometric analysis of Stat3 expression in cKD5 cells showed in SI.8.


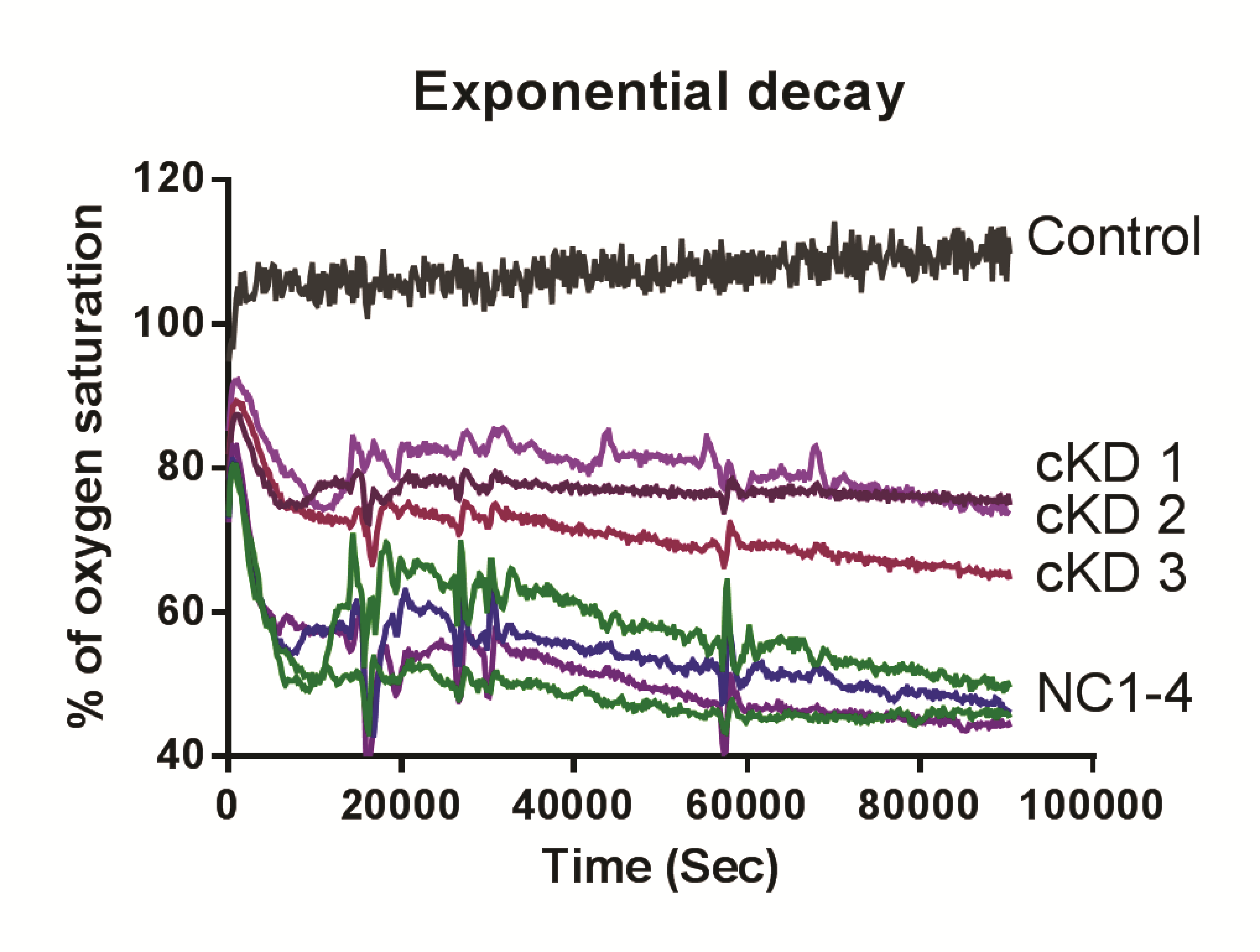


SI. 10: Comparison of oxygen saturation between cKD5 (three independent measurements) and NC cells (4 independent measurements)


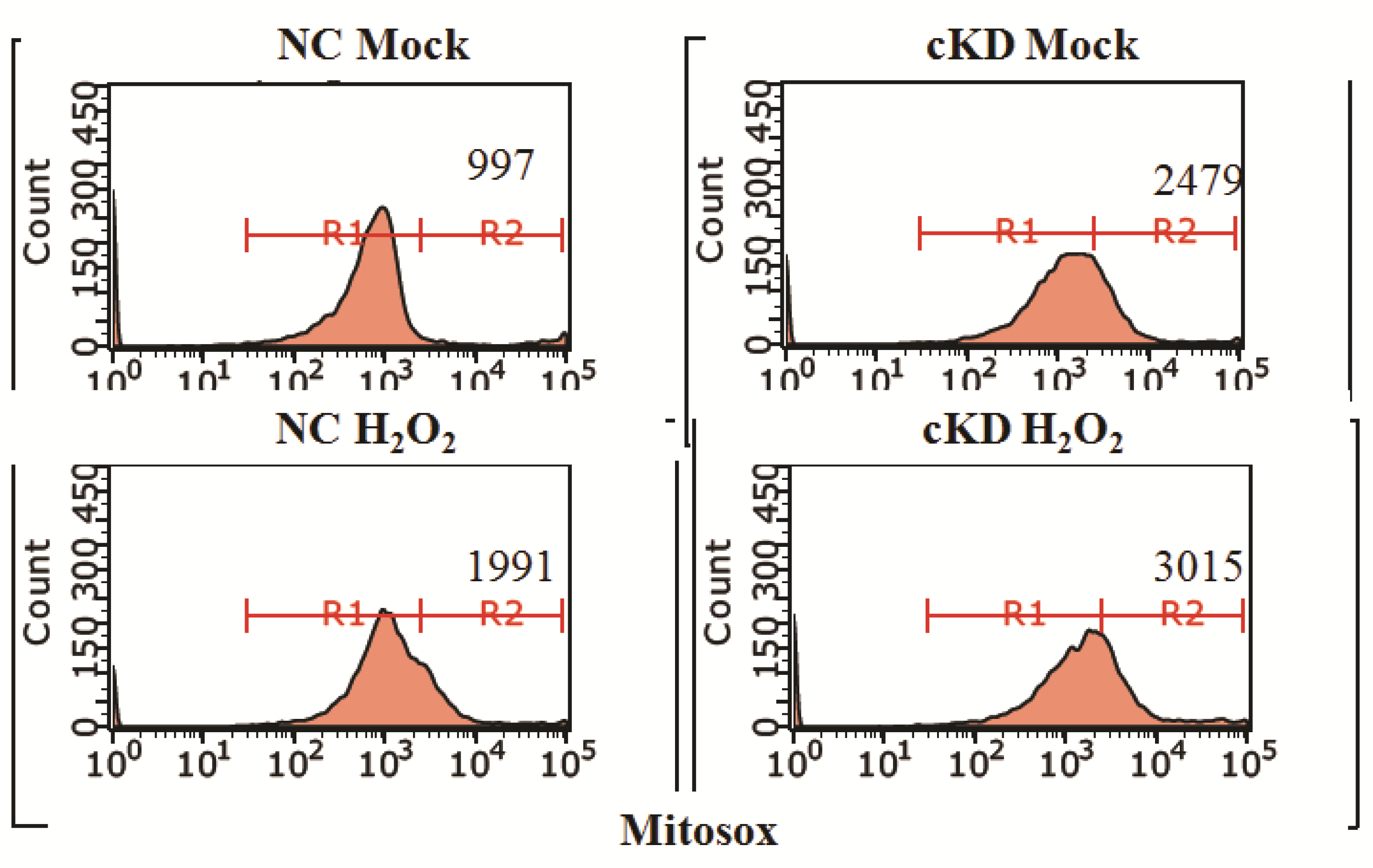


SI. 11: Fluorescent intensity of Mitosox in NC and cKD cells treated with H_2_O_2_.


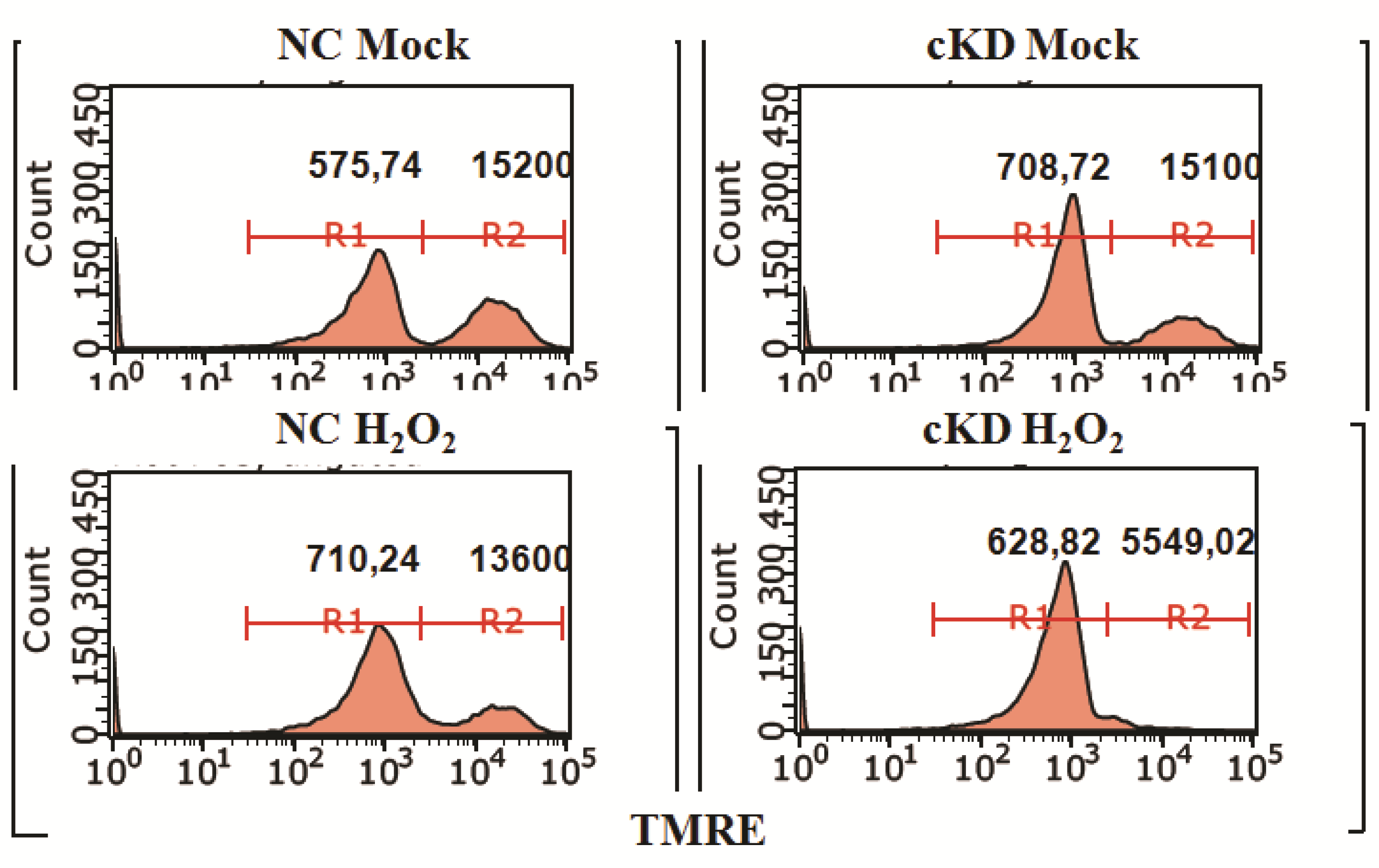


SI. 12: Fluorescent intensity of TMRE in NC and cKD cells treated with H_2_O_2_.


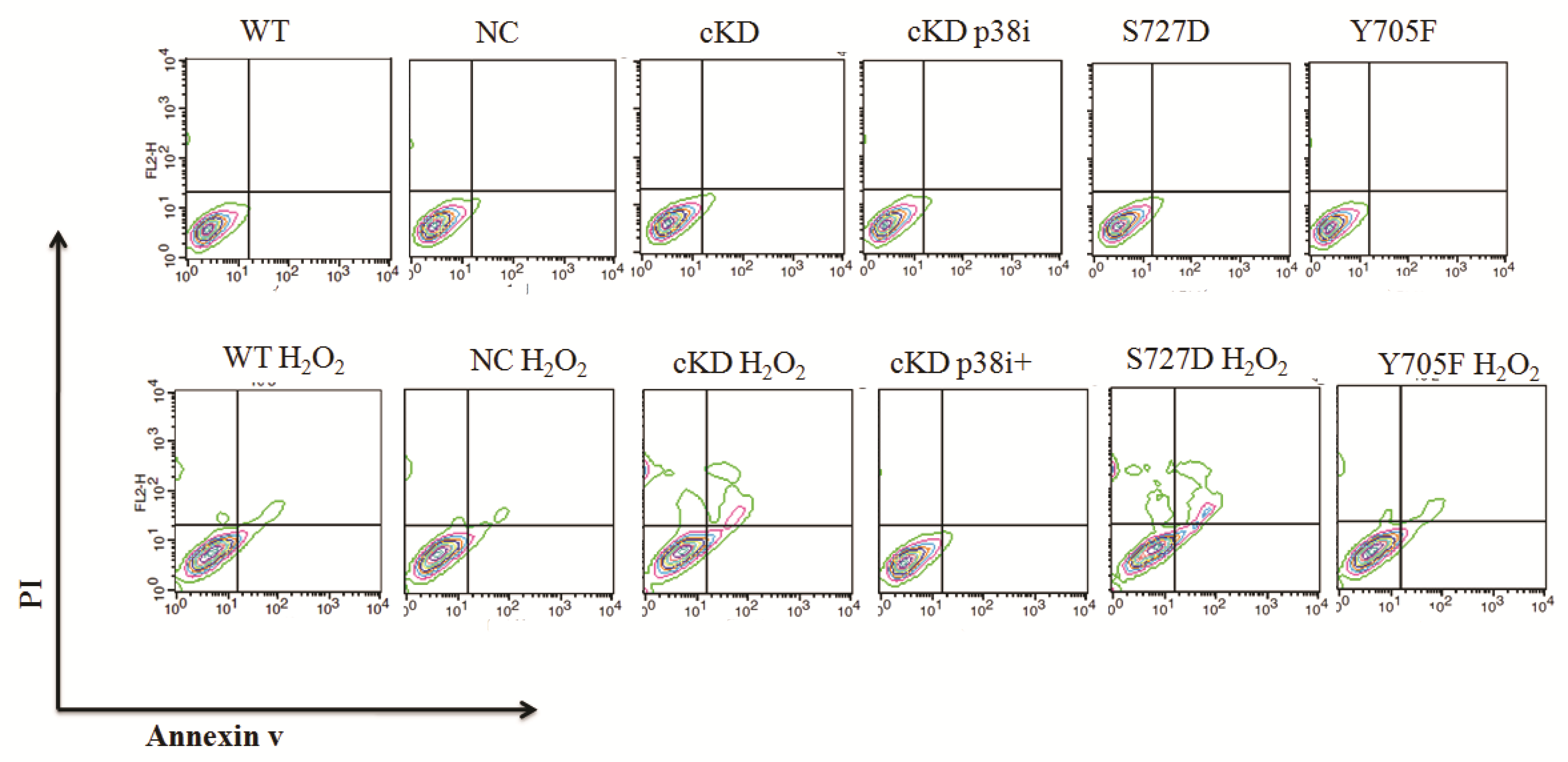


SI. 13: Dot plots of annexin v assay described in Fig. 1H.

SI. 14: Densitometric analysis of cleaved PARP in cKD5 cells showed in Fig. 3I.


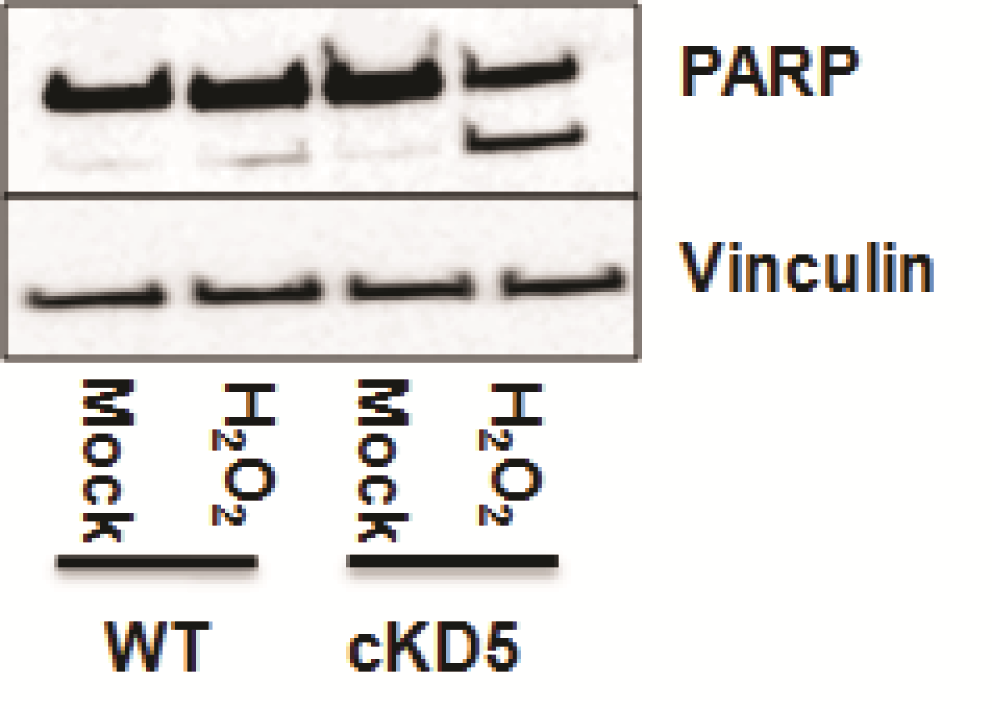


SI. 15: cKD5 cells are more sensitive to H_2_O_2_ treatment as compared to WT-

SI. 16: Densitometric analysis of mitochondrial and cytosolic Stat3 i showed in Fig. 3K.


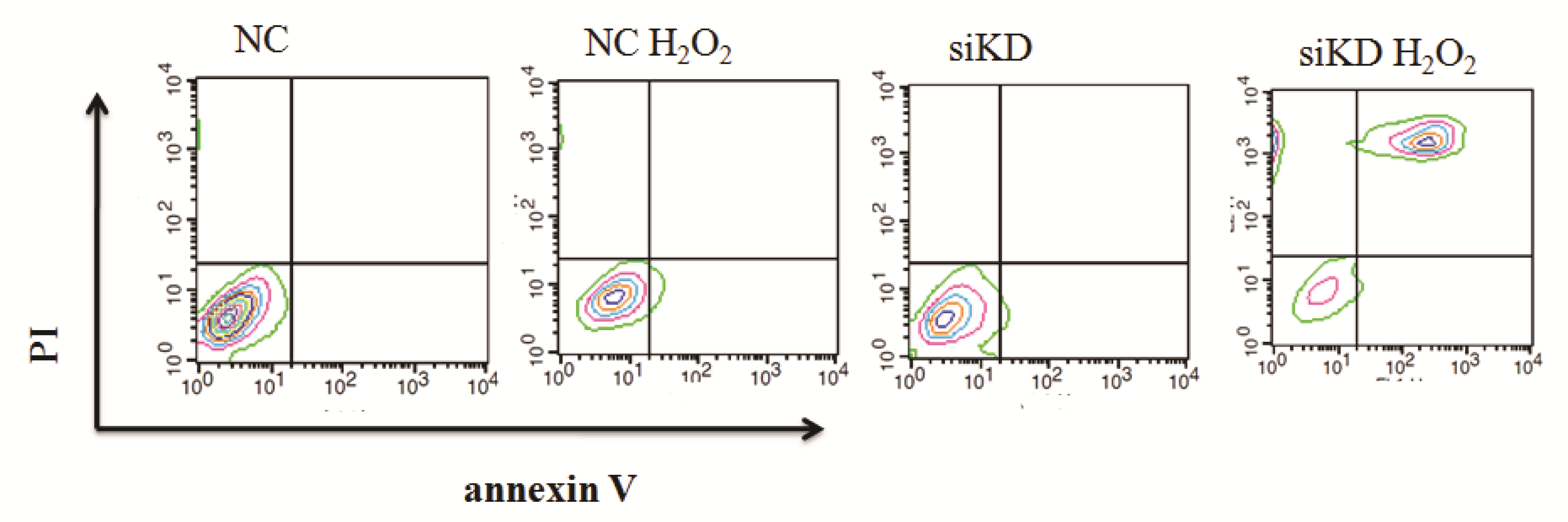


SI. 17: Dot plots of annexin v assay described in Fig. 4A


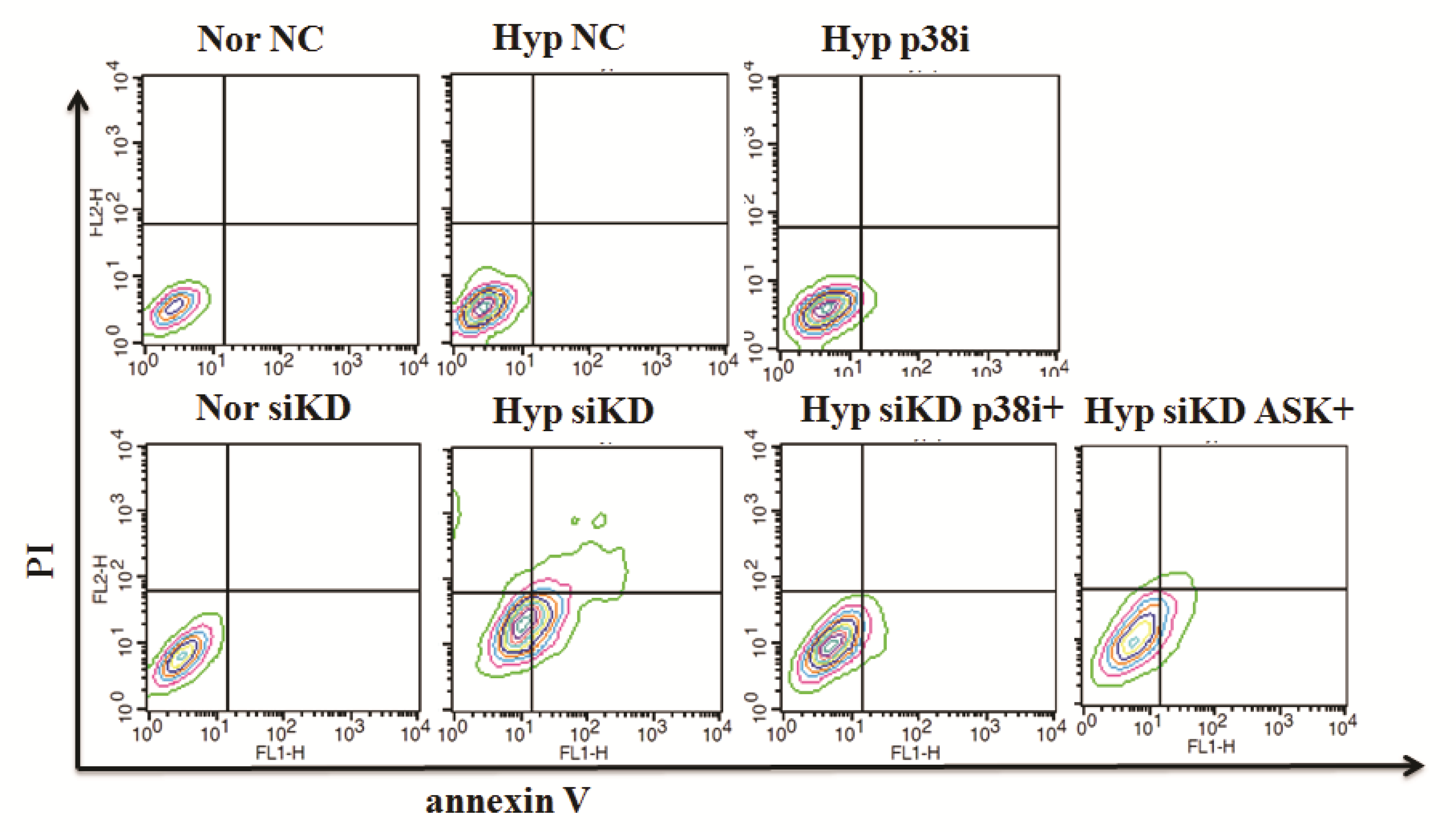


SI. 18: Dot plots of annexin v assay described in Fig. 4F.
